# Analysis of the Inter-Scale Agreement of Questionnaires to Assess the Emotional Eating in a Population of Polish Girls: PLACE-19 Study

**DOI:** 10.3390/nu18030457

**Published:** 2026-01-30

**Authors:** Dominika Guzek, Dominika Skolmowska, Dominika Głąbska

**Affiliations:** 1 Department of Food Market and Consumer Research, Institute of Human Nutrition Sciences, Warsaw University of Life Sciences (SGGW-WULS), 159C Nowoursynowska Street, 02-776 Warsaw, Poland; dominika_guzek@sggw.edu.pl; 2 Department of Dietetics, Institute of Human Nutrition Sciences, Warsaw University of Life Sciences (SGGW-WULS), 159C Nowoursynowska Street, 02-776 Warsaw, Poland; dominika_skolmowska@sggw.edu.pl

**Keywords:** emotional eating, adolescent, women, surveys and questionnaires, self-report, PLACE-19 Study

## Abstract

**Background/Objectives**: Emotional eating is a tendency to increase food consumption in order to modify a negative emotional state, but little is known about this phenomenon or about the way to assess it. The aim of the study was to assess the emotional eating in the population of Polish girls using various questionnaires, in order to compare the results obtained while using various tools. **Methods**: The emotional eating was assessed in a sample of *n* = 771 girls (age 15–18 years) from a nationwide PLACE-19 Study population, recruited based on a random quota sampling of Polish secondary schools. The results were gathered within the Computer-Assisted Web Interview (CAWI) and compared for four tools: Emotional Eating Scale for Children and adolescents (EES-C), Emotional Eating Subscale (EE-3) of Three-Factor Eating Questionnaire (TFEQ), Emotional Overeating Questionnaire (EOQ-5), and Emotional Eater Questionnaire (EEQ). **Results**: The analysis of correlation revealed statistically significant association for comparison of all the questionnaires (*p* < 0.001). For a comparison of EE-3 and EOQ-5, the strongest correlation between the results, and the second highest share of the studied group presenting agreement in emotional eating category was stated, indicating the strongest agreement. For a comparison of EES-C and EOQ-5, as well as EES-C and EEQ, the weakest correlation between the results, and the lowest share of the studied group presenting agreement in emotional eating category was stated, indicating the weakest agreement. **Conclusions**: It may be suggested that in studies conducted in a group of girls the recommended approach would be to use at least two various questionnaires to assess emotional eating, to enable comparing results, as the knowledge gathered so far does not allow an indication of the most reliable tool. As there is only a fair agreement between compared tools, the arbitrary choice of one tool may significantly influence the formulated conclusions. Moreover, there is a need to conduct more studies assessing emotional eating while using various questionnaire methods, in order to compare the results obtained while using various tools and to indicate the most reliable questionnaires.

## 1. Introduction

Emotional eating is a term introduced to describe a tendency to consume food in response to emotional states, rather than in response to physical hunger, in order to modify a negative emotional state [1]. In general, emotional eating may result from negative or positive emotions, but the majority of authors traditionally focus on negative emotions [2]. Taking this into account, some authors indicate also negative emotional eating, defined as overeating in response to negative emotions [3], and positive emotional eating, defined as overeating in response to positive emotions [4]. However, at the same time they indicate that those may be different constructs, and phenomena of positive emotional eating may be based on different mechanisms, being not a result of emotion regulation difficulties and interoceptive awareness, but rather of hedonic and external hunger influences [4]. As a result, emotional eating is often equated with negative emotional eating, and it is indicated that it is associated with a consumption of food products being palatable, energy-dense, and unhealthy in order to cope with emotions and states such as stress, anxiety, loneliness, or depression [4]. In contrary, positive emotional eating is associated with consumption of healthy products and positive eating habits [5].

Emotional eating is in general associated with a risk of increased body mass [6], and it may be link between emotional stress and increased food intake, leading to overweight or obesity [7]. Taking into account the increasing role of stress in modern societies [8], it is indicated by the World Health Organization (WHO) as the epidemic of the twenty-first century [9]. At the same time, WHO emphasizes the rising problem resulting from increasing body mass, as since 1990 adult obesity has more than doubled and adolescent obesity has quadrupled [10]. Considering those facts, managing emotional eating is becoming important, and a recent systematic review and meta-analysis by Smith et al. [11] indicated that body mass reduction interventions addressing emotional eating of patients are a promising tool in body mass reduction.

In spite of the fact that still little is known about emotional eating, it may be indicated that this phenomenon is gender-dependent, ethnicity-dependent, and age-dependent. Within the Family Health Habits Survey, which is a United States national study [12], it was indicated that the highest level of emotional eating was observed for female, Caucasians (but non-Hispanic), and younger individuals. Similarly, in a United States study conducted for adolescents, it was indicated that for girls there is an association between perceived stress, worries or tension/anxiety, and their emotional eating, while for boys, it was observed only for a confused mood [13]. At the same time, adolescence and early adulthood are indicated as periods with the highest level of emotional eating, as it increases since early adolescence [14], while in adults it decreases with age [15]. It was confirmed within a population from the Northern Finland Birth Cohort 1986 (NFBC1986), studied at the age of 16 years, that emotional eating was experienced by 43% of girls and by 15% of boys [16].

The appearance of emotional eating may be assessed while using various self-report questionnaires, addressed for various populations of adults and/or minors, and translated into various languages, including the following most important examples of questionnaires: Dutch Eating Behavior Questionnaire (DEBQ) [17], Emotional Eating Scale (EES) [18], Emotional Eating Scale for Children and adolescents (EES-C) [19], Three-Factor Eating Questionnaire (TFEQ) [20] with its Emotional Eating subscale (EE-3) [21], Emotional Appetite Questionnaire (EMAQ) [22], Emotional Overeating Questionnaire (EOQ) [23], used also as a shortened version of EOQ-5 [24], and Emotional Eater Questionnaire (EEQ) [25]. The design of the questionnaires is similar, as they are based on a questions or statements associated with emotional eating behaviors to be assessed if they are true or false in case of the responding individual [26]. However, still little is known about the phenomenon of emotional eating [27], and about the way to assess this phenomenon, as it is indicated that not only the results obtained using various questionnaires may differ, but also they may not be transferred into real-conditions behaviors [28].

In spite of the fact that the studies of emotional eating were traditionally focused on a female population [29], currently both female and male populations are assessed, but there are some gender-dependent challenges associated with questionnaire performance. For EES-C, some factorial variances across adolescent males and females were observed, while, depending on gender, the questionnaire seemed to measure different constructs for two items: worried and bored [30]. Moreover, some validation studies were conducted only for female populations, so extrapolation of the results on male populations may be constricted, which may be indicated for some studies conducted for DEBQ [31], EES [32,33], or TFEQ [34].

Taking this into account, the aim of the study was to assess emotional eating in the population of Polish girls using various questionnaires, in order to compare the results obtained while using various tools. It was hypothesized that the results obtained using different questionnaires would not differ, namely that regardless of the tool chosen, the same share of emotional eaters would be indicated, and that the level of emotional eating in individual respondents would not differ.

## 2. Materials and Methods

### 2.1. General Information

The data for the study were obtained within the PLACE-19 Study (Polish Adolescents’ COVID-19 Experience Study) in its 3rd phase (from 21 January to 17 February 2021), which focused on the psychological aspects of eating behaviors. Within the 3rd phase of the PLACE-19 Study, 4 questionnaires to assess emotional eating were applied: EES-C [35], EE-3 [36], EOQ-5 [37], and EEQ [38], but the analyses were presented for various sub-groups and the results obtained for various tools were not compared.

The PLACE-19 Study was conducted in the Institute of Human Nutrition Sciences, Warsaw University of Life Sciences (WULS-SGGW), on the basis of the approval of the Ethics Committee of the Central Clinical Hospital of the Ministry of Interior and Administration in Warsaw (No. 2/2021). The previous phases of the PLACE-19 Study focused on hygienic and personal protective behaviors (1st phase), as well as nutritional behaviors (2nd phase). All the procedures within the study were conducted in agreement with the Declaration of Helsinki, while participants and their parents/legal guardians expressed informed consent for the study participation and all the procedures.

### 2.2. Studied Population of Polish Girls

The presented analysis was conducted in the sub-population of the PLACE-19 Study participants, including only minor girls (aged < 18 years), in order to obtain a homogenic population, while in the PLACE-19 Study the general population included both female and male individuals in the age of 15–20 years (typical age for a secondary school students in Poland). The decision to study only the group of minor individuals results from the fact that education in Poland is compulsory until the age of 18 years [39]. Moreover, within the population gathered within the 3rd phase of the PLACE-19 Study, only a very small number represented the age of 19 and 20 years old (for female respondents 4.5% and 0.9% of the study population, respectively), which meant that the population gathered for these age groups failed to be representative of the national population.

The recruitment for the PLACE-19 Study was based on the random quota sampling of Polish secondary schools, due to the relatively high Net Enrollment Rate (NER) in Poland for secondary education (estimated as 89.38% based on the data of the Central Statistical Office (CSO) in Poland for December 2021 [40]). The recruitment was based on the national secondary school register, while for each phase of the study it was conducted separately.

As Poland is divided into 16 voivodeships (basic administrative unit of Poland), and each of them is divided into counties, the sampling was conducted to select (1) 5 counties from each voivodeship (resulting in 80 counties randomly selected), and (2) 5 secondary schools from each previously selected county (resulting in 400 secondary schools randomly selected). Each secondary school selected for the study was invited to participate by informing its principal, and if he agreed, the students and their parents/legal guardians were invited, and if they agreed their informed consents were gathered. The students providing informed consent received the link to the electronic version of the study questionnaire, as the Computer-Assisted Web Interview (CAWI) was conducted.

For the 3rd phase of the PLACE-19 Study, the following inclusion criteria were planned: students aged 15–20 years; students of the secondary schools randomly chosen for this phase of the PLACE-19 Study; and students providing their informed consent and the informed consent of their parents/legal guardians (for minor individuals).

For the 3rd phase of the PLACE-19 Study, the following exclusion criteria were planned: students participating in any previous phase of the PLACE-19 Study and students with any missing/unreliable data in the completed questionnaires.

Moreover, for the presented analysis, in order to obtain the homogenous population including only minor girls, the following additional exclusion criteria were planned: male students and students aged 18–20 years.

Based on the described procedure, the final sub-sample of minor girls gathered for the study included *n* = 771 individuals. In order to present a broader perspective, the same analysis as conducted for minor girls was also conducted for a total population of girls aged 15–20 and boys aged 15–20 and is presented in the Supplementary Materials.

### 2.3. Questionnaires Applied Within the Study

The tools applied for the presented analysis included 4 independent questionnaires to assess emotional eating: EES-C, EE-3, EOQ-5, and EEQ, with each of them separately presented and preceded by separate instructions in order to reduce the risk of transferring answers from one questionnaire to the following one.

EES-C was based on EES previously developed by Arnow et al. [18], while EES-C was adapted for use in 8–17-year-old children and validated by Tanofsky-Kraff et al. [19]. EES-C is based on a list of 25 emotions, which may be aggregated within major groups, as follows: anger (angry, jealous, furious, irritated, resentful, rebellious), anxiety (worried, upset, frustrated, nervous), depression (lonely, inadequate, blue, discouraged, bored, guilty, helpless, confused, sad), and somatic (jittery, shaky, on edge, uneasy, excited, worn out). For each emotion, a respondent is asked about her desire to eat in response to this emotion, while using a 5-point scale, as follows: ‘no desire’ (0 points), ‘small desire’ (1 point), ‘moderate desire’ (2 points), ‘strong desire’ (3 points), and ‘overwhelming urge to eat’ (4 points) [18]. The total emotional eating score is calculated based on 25 individual results for emotions, which is a value from a range of 0–100 [41] and was interpreted for cut-off of the median value [42], while for the obtained results the median was 25, so the results were interpreted as follows: (1) score ≤ 25—low emotional eating level and (2) score > 25—high emotional eating level. For the supplementary analysis for a sub-sample of boys, a median of 27 was observed, so the corresponding cut-off was applied.

EE-3 [21] emerged as a sub-scale of TFEQ, which was developed and validated by Stunkard & Messick [20]. EE-3 is based on a list of 3 statements, as follows: (1) ‘When I feel anxious, I find myself eating’, (2) ‘When I feel blue, I often overeat’, and (3) ‘When I feel lonely, I console myself by eating’. For each statement, a respondent is asked for a true or false response, using a 4-point scale, as follows: ‘definitely true’ (4 points), ‘mostly true’ (3 points), ‘mostly false’ (2 points), and ‘definitely false’ (1 point) [21]. The total emotional eating score is calculated as the sum of scores for individual statements minus 3 (being the lowest possible result), divided by 9 (being the possible score range), and multiplied by 100% [43]. It was interpreted as in the study by Oikarinen et al. [44], for cut-off of 55.6, as follows: (1) score < 55.6—low emotional eating level and (2) score ≥ 55.6—high emotional eating level.

EOQ was presented by Masheb and Grilo [23] as a 6-item scale including 5 negative and 1 positive emotions, while the other authors validated also a shortened version of EOQ-5, including 5 negative emotions only [24]. EOQ-5 is based on a list of 5 emotions with additional descriptions about how a person feels while experiencing a given emotion, as follows: anxiety (worry, jittery, nervous), sadness (blue, down, depressed), loneliness (bored, discouraged, worthless), tiredness (worn-out, fatigued), and anger (upset, frustrated, furious). For each emotion, a respondent is asked about how many days during the past 4 weeks (28 days) she has eaten an unusually large amount of food in response to this emotion, while using a 7-point scale, as follows: 0 days (0 points), 1–5 days (1 point), 6–12 days (2 points), 13–15 days (3 points), 16–22 days (4 points), 23–27 days (5 points), and 28 days (6 points) [23]. The total emotional eating score is calculated as a sum of 5 individual results for emotions, which is a value from a range of 0–30 and was interpreted for a cut-off of 2, as follows: (1) score ≤ 2—low emotional eating level and (2) score > 2—high emotional eating level, based on the cut-off in the study by Casu et al. [24], being the median value in the referred study, as well as in the presented study group.

EEQ was developed and validated by Garaulet et al. [25]. EEQ is based on a list of 10 questions, as follows: (1) ‘Do the weight scales have a great power over you? Can they change your mood?’, (2) ‘Do you crave specific foods?’, (3) ‘Is it difficult for you to stop eating sweet things, especially chocolate?’, (4) ‘Do you have problems controlling the amount of certain types of food you eat?’, (5) ‘Do you eat when you are stressed, angry or bored?’, (6) ‘Do you eat more of your favorite food and with less control when you are alone?’, (7) ‘Do you feel guilty when eat “forbidden” foods, like sweets or snacks?’, (8) ‘Do you feel less control over your diet when you are tired after work at night?’, (9) ‘When you overeat while on a diet, do you give up and start eating without control, particularly food that you think is fattening?’, and (10) ‘How often do you feel that food controls you, rather than you controlling food?’. For each question, a respondent is asked about the extent to which her eating behaviors are affected by emotions, while using a 4-point scale, as follows: ‘never’ (0 points), ‘sometimes’ (1 point), ‘generally’ (2 points), and ‘always’ (3 points). The total emotional eating score is calculated as the sum of 10 individual results for questions, which was a value from a range of 0–30 and was interpreted as follows: (1) score ≤ 10—low emotional eating level (non-emotional eater/low emotional eater) and (2) score > 10—high emotional eating level (emotional eater/very emotional eater) [25].

The questionnaires were adopted for the Polish population according to the recommendations by WHO [45], in a 3-step procedure. The procedure included (1) forward translation from English to Polish, conducted by a Polish researcher being fluent in English, (2) backward translation from Polish to English, conducted by a Polish researcher being fluent in English (but different than in the 1st step), and (3) final polishing, conducted by the panel of experts, being Polish researchers fluent in English (but different than in the 1st and 2nd steps).

Additionally, the questionnaire included questions allowing verification of the inclusion and exclusion criteria, as well as allowing the body mass assessment. Based on the declared body weight and height, the Body Mass Index (BMI) was calculated and interpreted based on the Polish OLAF growth charts [46], based on the gender- and age-specific growth reference values for Polish children [47]. The following cut-offs by WHO were applied: (1) <5th percentile—underweight, (2) 5th–85th percentile—normal body weight, (3) 85th–95th percentile—overweight, and (4) >95th percentile—obesity [48].

### 2.4. Statistical Analysis

The normality of distribution was interpreted using the Shapiro–Wilk test. To compare sub-groups, the *t*-Student test and Mann–Whitney test were used based on the data distribution (for constant variables), or the chi^2^ test with Yates’ correction if needed (for categorical variables). To verify correlations, Spearman’s rank correlation coefficient was applied. To verify the internal reliability of data, Cronbach’s α and McDonald’s ω coefficients were applied. To compare results obtained for various tools, the following tests were applied: Cohen’s kappa, and McNemar’s test.

*p* ≤ 0.05 was considered statistically significant. The statistical analysis was conducted using Statistica 13.0 (Statsoft Inc., Tulsa, OK, USA) and Jamovi (the Jamovi project, version 2.6.44, Sydney, Australia).

## 3. Results

### 3.1. Baseline Information

The general characteristics of the group of girls studied within the PLACE-19 Study are presented in Table 1. The majority of the studied girls were of a normal body weight (76%), while one-fifth had excessive body weight (20.9%), and some of them were also underweight (3.1%).

The results of Cronbach’s α and McDonald’s ω coefficients for various questionnaire methods in the group of girls studied within the PLACE-19 Study are presented in Table 2. For both Cronbach’s α and McDonald’s ω, the highest values were observed for EES-C (0.95, and 0.945, respectively), while for the other questionnaires the results were comparable.

The summary of the results obtained for emotional eating gathered in the group of girls studied within the PLACE-19 Study using various questionnaire methods is presented in Table 3. Due to various ranges for various questionnaires, the raw results are incomparable.

The share of individuals classified as emotional eaters and not emotional eaters in the group of girls studied within the PLACE-19 Study using various questionnaire methods is presented in Table 4. The lowest share of the studied group was classified as emotional eaters for EE-3 (31.6%), while the highest was for EES-C (50.6%).

**Figure 1:**
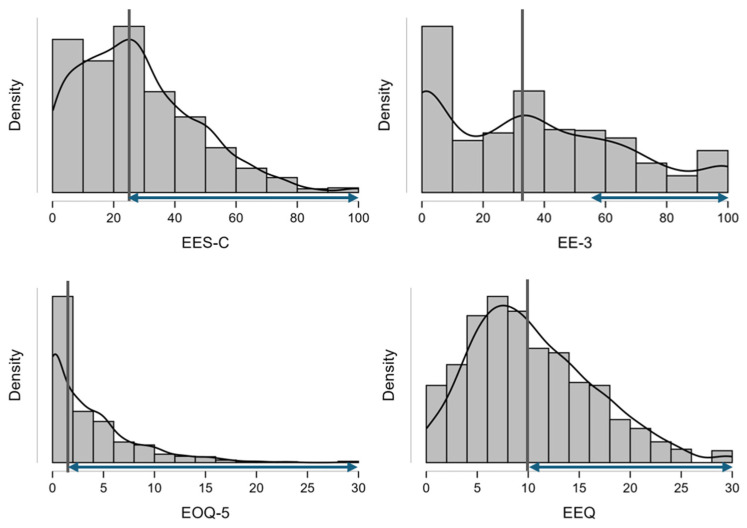
Histogram analysis of emotional eating level assessed in the group of girls studied within the PLACE-19 Study using various questionnaire methods (median values marked with black lines; values attributed to emotional eaters, based on cut-off values, marked with blue arrows); EES-C—Emotional Eating Scale for Children and adolescents; EOQ-5—Emotional Overeating Questionnaire; EE-3—Emotional Eating scale; EEQ—Emotional Eater Questionnaire.

The histogram analysis of emotional eating level assessed in the group of girls studied within the PLACE-19 Study using various questionnaire methods is presented in Figure 1. The histograms present various scales, accompanied by medians, as well as values attributed to emotional eaters based on cut-offs.

### 3.2. Inter-Scale Agreement

The analysis of correlation of emotional eating level assessed in the group of girls studied within the PLACE-19 Study using various questionnaire methods is presented in Table 5. The analysis of correlation revealed a statistically significant association for comparison of all the questionnaires (*p* < 0.001), with the strongest being for the comparison of EE-3 and EOQ-5 (ρ = 0.50), and the weakest for the comparison of EES-C and EEQ (ρ = 0.32). The analysis of correlation of emotional eating level assessed in the group of girls and boys studied within the PLACE-19 Study, aged 15–20, using various questionnaire methods, is presented in Supplementary Table S1 and Supplementary Table S2, respectively.

The analysis of inter-scale agreement based on discordance (%) between various questionnaire methods for the classification into emotional eating categories in the group of girls studied within the PLACE-19 Study is presented in Table 6. The highest share of the studied group classified oppositely (as emotional eaters or as not emotional eaters), depending on the method, was observed for comparison of EES-C with other methods (36.4–38.4%). For all other comparisons, about one-third of individuals were classified oppositely depending on the method (33.1–33.6%). The analysis of inter-scale agreement based on discordance (%) between various questionnaire methods for the classification into emotional eating categories in the group of girls and boys studied within the PLACE-19 Study, aged 15–20, is presented in Supplementary Table S3 and Supplementary Table S4, respectively.

The analysis of inter-scale agreement based on Cohen’s kappa for comparison between various questionnaire methods for the classification into emotional eating categories in the group of girls studied within the PLACE-19 Study is presented in Table 7. For all inter-scale comparisons, Cohen’s kappa indicated a fair agreement. The analysis of inter-scale agreement based on Cohen’s kappa for comparison between various questionnaire methods for the classification into emotional eating categories in the group of girls and boys studied within the PLACE-19 Study, aged 15–20, is presented in Supplementary Table S5 and Supplementary Table S6, respectively.

The analysis of inter-scale agreement based on McNemar’s test with test matrix data for comparison between various questionnaire methods for the classification into emotional eating categories in the group of girls studied within the PLACE-19 study is presented in Table 8. Based on McNemar’s test, only for a comparison of EES-C and EOQ-5, an inter-scale agreement may be questioned (*p* = 0.7713). The EES-C questionnaire more often classified individuals as emotional eaters than all other questionnaires (EE-3, EOQ-5, EEQ), and the EOQ-5 questionnaire classified individuals as emotional eaters more often than the EEQ questionnaire. The EE-3 questionnaire classified individuals as emotional eaters less often than EOQ-5 and EEQ questionnaires. The inter-scale agreement based on McNemar’s test with test matrix data for comparison between various questionnaire methods for the classification into emotional eating categories in the group of girls and boys studied within the PLACE-19 Study, aged 15–20, is presented in Supplementary Table S7 and Supplementary Table S8, respectively.

### 3.3. BMI-Stratified Comparisons

The summary of the results obtained for emotional eating gathered in the group of girls studied within the PLACE-19 study using various questionnaire methods, stratified by BMI, is presented in Table 9. It was observed that the emotional eating results gathered while using EE-3 (*p* = 0.004) and EEQ differed between sub-samples of normal body weight and underweight individuals (*p* < 0.001), when compared with the sub-sample of excessive body mass individuals. For both questionnaires, a higher emotional eating level was observed for excessive body mass individuals.

The share of individuals classified as emotional eaters and not emotional eaters, in the group of girls studied within the PLACE-19 Study, using various questionnaire methods stratified by BMI, is presented in Table 10. It was observed that only for EEQ did the share of emotional eaters and not emotional eaters differ between sub-sample of normal body weight and underweight individuals, while compared with sub-sample of excessive body mass individuals (*p* < 0.001) and for excessive body mass individuals a higher share of emotional eaters was observed.

## 4. Discussion

The comparison of the questionnaires applied for the assessment of emotional eating allows an indication of which tools lead to the formulation of similar conclusions, but it does not allow one questionnaire to be stated as being better than the others. This result is due to various reasons, with the most important one associated with the fact that there is no standardization or methodological gold standard for the assessment of emotional eating [49]. Moreover, it is commonly indicated that the questionnaire alone does not allow the assessment of emotional eating in depth, so lab-based assessments and ecological momentary assessment (EMA) should be additionally included [28]. Without it, no one can be sure that the questionnaires actually measure what they are intended to, namely increased consumption resulting from specific emotions (not the declaration of the increased consumption formulated based on subjective assessment) [26]. Based on this assumption, some authors [49], searching for a gold standard, rather indicate a so-called bogus ‘taste’ test, for which individuals are sure that they only give taste ratings of various food products, but their actual food intake is unobtrusively recorded [50]. Lack of a gold standard and uncertainty about what is actually measured while using the questionnaires to assess emotional eating are also associated with the lack of a unified definition of emotional eating [5], as well as the fact that still little is known about this phenomenon and there are a lot of controversies [49].

From comparing the studied methods, it may be noted that there are some pairs of methods giving similar results independently from the method of comparison. This was noted for a comparison of EE-3 and EOQ-5, with the strongest correlation between the results, and the second highest share of the studied group presenting agreement in the emotional eating category. At the same time, the weakest associations were stated for the pairs of EES-C and EOQ-5, as well as EES-C and EEQ, based on the weakest correlation between the results, and the lowest share of the studied group presenting agreement in the emotional eating category. Moreover, this observation corresponds with the results of the agreement between various questionnaire methods for the classification into emotional eating categories, as for all the pairs a fair agreement was stated, except for the pair of EES-C and EOQ-5. The difference of EES-C may be explained by the analysis of the share of individuals classified as emotional eaters and not emotional eaters, as the results of EES-C indicated a significantly higher share of emotional eaters (51%) than for the other methods (32–49%).

Moreover, it should be indicated that, depending on the questionnaire, various associations with BMI were stated. The differences of both emotional eating level and frequency between sub-samples of normal body weight and underweight individuals when compared with the sub-sample of excessive body mass individuals were stated for EEQ, while for EE-3 there was a difference only for emotional eating level, but not for frequency. This may allow one to suppose that various tools may be characterized by various sensitivity, depending on body mass; however, this requires further studies.

The difference of the results of EES-C in comparison with other methods corresponds to the highest Cronbach’s α (0.95) and McDonald’s ω for this method (0.945), when compared with the other methods (Cronbach’s α: 0.84–0.85; McDonald’s ω: 0.841–0.848). McDonald’s ω is considered acceptable for values of at least 0.7 [51], which was obtained for all the verified questionnaires. At the same time, for Cronbach’s α it is indicated that the acceptable values range from 0.7 to 0.95 [52], and some authors even recommend its value to be not higher than 0.9; as it is a measure of the internal consistency of a test or scale, too high values suggest that some questions should be removed, as they test the same issue but in a different way [53].

There were only a few other studies comparing methods to assess emotional eating, but none of them included four various questionnaires. In the study by Ghafouri et al. [54], EEQ was compared with Salzburg Emotional Eating Scale (SEES), and the study indicated R of 0.36 for correlation, being similar to the presented study for EEQ. In the study by Schnepper et al. [28], DEBQ was compared with SEES, and for relevant subscales, R of 0.491 for correlation was stated, being a value comparable with the strongest correlation in the presented study.

Compared with the other studies [28,54], it may be indicated that the presented study conducted in a group of girls not only analyzed more questionnaires in a larger group of respondents, being population-based, but it presented a weaker agreement of the compared questionnaires. Taking this into account, it may be indicated that an arbitrary choice of any specific questionnaire by researcher may lead to a questionnaire-related bias, so a fair agreement of the questionnaires implies rather a multiple scale use. At the same time, the practical scale selection criteria may not only be based on administration length and convenience, but the reliability and construct should be taken into account.

Taking this into account, it should be indicated that there is a need to conduct more studies assessing emotional eating while using various questionnaire methods, as well as using the other methods, such as lab-based assessments and EMA, in order to compare the results obtained while using various methods and to indicate the most reliable questionnaires. As the conducted study is to our best knowledge the first one assessing the agreement between compared questionnaires, its results should be verified in other populations. Moreover, as the conducted study indicated only a fair agreement, it should be taken into account while choosing a questionnaire to assess emotional eating.

However, limitations of this study should also be indicated. First of all, using the questionnaires in the assessment of emotional eating, as for the other questionnaires, is always associated with a typical recall bias, resulting from gathering self-reported data, which may be even higher in the case of adolescents due to a social desirability bias. This results from the fact that the questionnaires used are within the same group of methods of emotional eating assessment, which also have limitations associated with the applied cut-offs; for EES-C, the median-based cut-off, being specific for a studied population, was used, but for the other questionnaires cut-offs were elaborated by other authors. Moreover, the study was conducted during the COVID-19 pandemic, which is a well-known factor that influenced nutritional behaviors, potentially including emotional eating. As a result, it may have also influenced the associations between emotional eating questionnaire responses.

## 5. Conclusions

It may be suggested that in studies conducted in a group of girls, the recommendation would be to use at least two various questionnaires to assess emotional eating to enable the comparison of results, as the knowledge gathered so far does not allow an indication of the most reliable tool. As there is only a fair agreement between compared tools, the arbitrary choice of one tool may significantly influence the formulated conclusions. Moreover, there is a need to conduct more studies assessing emotional eating while using various questionnaire methods in order to compare the results obtained while using various tools and to indicate the most reliable questionnaires.

## Figures and Tables

**Table 1:** General characteristics of the group of girls studied within the PLACE-19 Study (*n* = 771).

Characteristics		Details	
		Mean ± SD	Median (IQR)
Age (years)		16.6 ± 1.0	17.0 * (1.0)
BMI interpretation based on the growth reference values (percentile)		54.6 ± 29.1	56.0 * (54.0)
		***n* (%)**	
Body mass	Underweight	24 (3.1%)	
	Normal body weight	586 (76.0%)	
	Overweight	100 (13.0%)	
	Obesity	61 (7.9%)	

* non-normal distribution (evidenced by Shapiro–Wilk test; *p* < 0.05); BMI—Body Mass Index; SD—Standard Deviation; IQR—Interquartile range.

**Table 2:** The results of Cronbach’s α and McDonald’s ω coefficients for various questionnaire methods in the group of girls studied within the PLACE-19 Study.

Questionnaire	Cronbach’s α	McDonald’s ω		
		McDonald’s ω	95% Lower Bound	95% Upper Bound
EES-C	0.95	0.945	0.939	0.951
EOQ-5	0.84	0.841	0.823	0.858
EE-3	0.84	0.845	0.826	0.864
EEQ	0.85	0.848	0.831	0.864

EES-C—Emotional Eating Scale for Children and adolescents; EOQ-5—Emotional Overeating Questionnaire; EE-3—Emotional Eating scale; EEQ—Emotional Eater Questionnaire.

**Table 3:** Summary of the results obtained for emotional eating gathered in the group of girls studied within the PLACE-19 Study using various questionnaire methods.

Questionnaire	Details	
	Mean ± SD	Median (IQR)
EES-C	27.6 ± 19.4	25.0 * (27.0)
EE-3	35.8 ± 30.7	33.3 * (55.6)
EOQ-5	3.8 ± 4.5	2.0 * (5.0)
EEQ	10.5 ± 6.1	10.0 * (8.0)

* non-normal distribution (evidenced by Shapiro–Wilk test; *p* < 0.05); EES-C—Emotional Eating Scale for Children and adolescents; EOQ-5—Emotional Overeating Questionnaire; EE-3—Emotional Eating scale; EEQ—Emotional Eater Questionnaire; SD—Standard Deviation; IQR—Interquartile range.

**Table 4:** Share of individuals classified as emotional eaters and not emotional eaters in the group of girls studied within the PLACE-19 Study using various questionnaire methods.

Questionnaire	Emotional Eater	Not Emotional Eater
EES-C	390 (50.6%)	381 (49.4%)
EE-3	244 (31.6%)	527 (68.4%)
EOQ-5	375 (48.6%)	396 (51.4%)
EEQ	340 (44.1%)	431 (55.9%)

EES-C—Emotional Eating Scale for Children and adolescents; EOQ-5—Emotional Overeating Questionnaire; EE-3—Emotional Eating scale; EEQ—Emotional Eater Questionnaire.

**Table 5:** Analysis of correlation (Spearman rank correlation coefficient) of emotional eating level assessed in the group of girls studied within the PLACE-19 Study using various questionnaire methods.

Questionnaire	Results	EES-C	EE-3	EOQ-5
EE-3	*p*	<0.001	-	
	ρ	0.46		
	95% CI	0.40–0.52		
EOQ-5	*p*	<0.001	<0.001	-
	ρ	0.37	0.50	
	95% CI	0.31–0.43	0.44–0.55	
EEQ	*p*	<0.001	<0.001	<0.001
	ρ	0.32	0.41	0.47
	95% CI	0.26–0.39	0.35–0.47	0.41–0.52

EES-C—Emotional Eating Scale for Children and adolescents; EOQ-5—Emotional Overeating Questionnaire; EE-3—Emotional Eating scale; EEQ—Emotional Eater Questionnaire; 95% CI—95% Confidence Interval for Spearman’s ρ coefficient.

**Table 6:** Analysis of inter-scale agreement based on discordance (%) between various questionnaire methods for the classification into emotional eating categories in the group of girls studied within the PLACE-19 Study.

Questionnaire	EES-C	EE-3	EOQ-5
EE-3	36.4	-	
EOQ-5	38.4	33.6	-
EEQ	37.7	33.6	33.1

EES-C—Emotional Eating Scale for Children and adolescents; EOQ-5—Emotional Overeating Questionnaire; EE-3—Emotional Eating scale; EEQ—Emotional Eater Questionnaire.

**Table 7:** Analysis of inter-scale agreement based on Cohen’s kappa (95% CI) for comparison between various questionnaire methods for the classification into emotional eating categories in the group of girls studied within the PLACE-19 Study (*p* < 0.001 for all the analysis).

Questionnaire	EES-C	EE-3	EOQ-5
EE-3	0.268 (0.204–0.332)	-	
EOQ-5	0.232 (0.163–0.301)	0.321 (0.258–0.385)	-
EEQ	0.244 (0.176–0.312)	0.273 (0.206–0.340)	0.336 (0.270–0.403)

EES-C—Emotional Eating Scale for Children and adolescents; EOQ-5—Emotional Overeating Questionnaire; EE-3—Emotional Eating scale; EEQ—Emotional Eater Questionnaire.

**Table 8:** Analysis of inter-scale agreement based on McNemar’s test with test matrix data for comparison between various questionnaire methods for the classification into emotional eating categories in the group of girls studied within the PLACE-19 study.

Questionnaire	EES-C			EE-3			EOQ-5		
	*p*	b (A+, B−)	c (A−, B+)	*p*	b (A+, B−)	c (A−, B+)	*p*	b (A+, B−)	c (A−, B+)
EE-3	0.0001	209	72	-	-	-			
EOQ-5	0.7713	151	145	<0.0001	64	195	-	-	-
EEQ	0.0190	166	125	<0.0001	86	182	0.0332	145	110

EES-C—Emotional Eating Scale for Children and adolescents; EOQ-5—Emotional Overeating Questionnaire; EE-3—Emotional Eating scale; EEQ—Emotional Eater Questionnaire; b (A+, B−)—number of participants classified as emotional eaters by questionnaire A and not emotional eaters by questionnaire B; c (A−, B+)—number of participants classified as not emotional eaters by questionnaire A and emotional eaters by questionnaire B.

**Table 9:** Summary of the results obtained for emotional eating gathered in the group of girls studied within the PLACE-19 Study using various questionnaire methods, stratified by Body Mass Index (BMI).

Questionnaire	BMI < 25 kg/m^2^ (*n* = 610)		BMI ≥ 25 kg/m^2^ (*n* = 161)		*p*
	Mean ± SD	Median (IQR)	Mean ± SD	Median (IQR)	
EES-C	27.1 ± 19.1	25 * (26.0)	29.7 ± 20.6	28 * (31.0)	0.162
EE-3	34.3 ± 30.7	33.3 * (55.6)	41.3 ± 30.1	44.4 * (55.6)	0.004
EOQ-5	3.6 ± 4.3	2 * (5.0)	4.4 ± 5.2	3 * (5.0)	0.109
EEQ	10.1 ± 6.0	9 * (8.0)	12.0 ± 6.3	12 * (10.0)	<0.001

* non-normal distribution (evidenced by Shapiro–Wilk test; *p* < 0.05); EES-C—Emotional Eating Scale for Children and adolescents; EOQ-5—Emotional Overeating Questionnaire; EE-3—Emotional Eating scale; EEQ—Emotional Eater Questionnaire; SD—Standard Deviation; IQR—Interquartile range.

**Table 10:** Share of individuals classified as emotional eaters and not emotional eaters in the group of girls studied within the PLACE-19 Study using various questionnaire methods, stratified by Body Mass Index (BMI) (χ^2^ test with Yates’ correction).

Questionnaire	BMI < 25 kg/m^2^ (*n* = 610)		BMI ≥ 25 kg/m^2^ (*n* = 161)		χ^2^ (*df*)	*p*
	Emotional Eater	Not Emotional Eater	Emotional Eater	Not Emotional Eater		
EES-C	290 (47.5%)	320 (52.5%)	91 (56.5%)	70 (43.5%)	3.76 (1)	0.0525
EE-3	184 (30.2%)	426 (69.8%)	60 (37.3%)	101 (62.7%)	2.65 (1)	0.1034
EOQ-5	290 (47.5%)	320 (52.5%)	85 (52.8%)	76 (47.2%)	1.21 (1)	0.2723
EEQ	250 (41%)	360 (59%)	90 (55.9%)	71 (44.1%)	10.90 (1)	<0.0001

EES-C—Emotional Eating Scale for Children and adolescents; EOQ-5—Emotional Overeating Questionnaire; EE-3—Emotional Eating scale; EEQ—Emotional Eater Questionnaire; χ^2^—chi-square; *df*—degrees of freedom.

## Data Availability

The original contributions presented in this study are included in the article/Supplementary Material. Further inquiries can be directed to the corresponding author.

## Supplementary Materials

The following supporting information can be downloaded at: https://www.mdpi.com/article/10.3390/nu18030457/s1, Supplementary Table S1: Analysis of correlation (Spearman rank correlation coefficient) of emotional eating level, assessed in the group of girls studied within the PLACE-19 Study, aged 15–20, using various questionnaire methods; Supplementary Table S2: Analysis of correlation (Spearman rank correlation coefficient) of emotional eating level, assessed in the group of boys studied within the PLACE-19 Study, aged 15–20, using various questionnaire methods; Supplementary Table S3: Analysis of inter-scale agreement based on discordance (%) between various questionnaire methods for the classification into emotional eating categories in the group of girls studied within the PLACE-19 Study, aged 15–20; Supplementary Table S4: Analysis of inter-scale agreement based on discordance (%) between various questionnaire methods for the classification into emotional eating categories in the group of boys studied within the PLACE-19 Study, aged 15–20; Supplementary Table S5: Analysis of inter-scale agreement based on Cohen’s kappa (95% CI) for comparison between various questionnaire methods for the classification into emotional eating categories in the group of girls studied within the PLACE-19 Study, aged 15–20; Supplementary Table S6: Analysis of inter-scale agreement based on Cohen’s kappa (95% CI) for comparison between various questionnaire methods for the classification into emotional eating categories in the group of boys studied within the PLACE-19 Study, aged 15–20; Supplementary Table S7: Analysis of inter-scale agreement based on McNemar’s test with test matrix data for comparison between various questionnaire methods for the classification into emotional eating categories in the group of girls studied within the PLACE-19 Study, aged 15–20; Supplementary Table S8: Analysis of inter-scale agreement based on McNemar’s test with test matrix data for comparison between various questionnaire methods for the classification into emotional eating categories in the group of boys studied within the PLACE-19 Study, aged 15–20.

## Abbreviations

The following abbreviations are used in this manuscript:

BMI	Body Mass Index
DEBQ	Dutch Eating Behavior Questionnaire
EE-3	Emotional Eating Scale
EES-C	Emotional Eating Scale for Children and adolescents
EEQ	Emotional Eater Questionnaire
EMA	Ecological Momentary Assessment
EOQ-5	Emotional Overeating Questionnaire 5-item
IQR	Interquartile range
SEES	Salzburg Emotional Eating Scale
SD	Standard Deviation
TFEQ	Three-Factor Eating Questionnaire
WHO	World Health Organization
