# Supplementary material for: Analysis of the Inter-Scale Agreement of Questionnaires to Assess the Emotional Eating in a Population of Polish Girls: PLACE-19 Study
Source: Nutrients. 2026 Jan 30;18(3):457. doi: 10.3390/nu18030457 (PMC12899171; doi:10.3390/nu18030457)
Supplement: Supplementary file 1 [file nutrients-18-00457-s001.zip › nutrients-4076656-supplementary.pdf]

Supplementary Materials

Analysis of the Inter-Scale Agreement of Questionnaires to Assess the Emotional Eating in a Population of Polish Girls: PLACE-19 Study

Dominika Guzek [1], Dominika Skolmowska [2] and Dominika Głąbska [2,*]

[1] Department of Food Market and Consumer Research, Institute of Human Nutrition Sciences, Warsaw University of Life Sciences (SGGW-WULS), 159C Nowoursynowska Street, 02-776 Warsaw, Poland; dominika_guzek@sggw.edu.pl
[2] Department of Dietetics, Institute of Human Nutrition Sciences, Warsaw University of Life Sciences (SGGW-WULS), 159C Nowoursynowska Street, 02-776 Warsaw, Poland; dominika_skolmowska@sggw.edu.pl
* Correspondence: dominika_glabska@sggw.edu.pl; Tel.: +48-22-593-71-26

Supplementary Table S1. Analysis of correlation (Spearman rank correlation coefficient) of emotional eating level, assessed in the group of girls studied within the PLACE-19 Study, aged 15-20, using various questionnaire methods.

Questionnaire	Results	EES-C	EE-3	EOQ-5
EE-3	*p*	<0.001	-	
	ϱ	0.46		
	95% CI	0.41-0.52		
EOQ-5	*p*	<0.001	<0.001	-
	ϱ	0.36	0.48	
	95% CI	0.30-0.42	0.43-0.53	
EEQ	*p*	<0.001	<0.001	<0.001
	ϱ	0.34	0.42	0.47
	95% CI	0.27-0.40	0.37-0.48	0.41-0.52

EES-C – Emotional Eating Scale for Children and adolescents; EOQ-5 – Emotional Overeating Questionnaire; EE-3 – Emotional Eating scale; EEQ – Emotional Eater Questionnaire; 95% CI – 95% Confidence Interval for the Spearman's ϱ coefficient.

Supplementary Table S2. Analysis of correlation (Spearman rank correlation coefficient) of emotional eating level, assessed in the group of boys studied within the PLACE-19 Study, aged 15-20, using various questionnaire methods.

Questionnaire	Results	EES-C	EE-3	EOQ-5
EE-3	*p*	<0.001	-	
	ϱ	0.19		
	95% CI	0.08-0.23		
EOQ-5	*p*	0.001	<0.001	-
	ϱ	0.18	0.40	
	95% CI	0.07-0.29	0.30-0.49	
EEQ	*p*	<0.001	<0.001	<0.001
	ϱ	0.23	0.39	0.48

95% CI	0.12-0.34	0.29-0.48	0.39-0.56

EES-C – Emotional Eating Scale for Children and adolescents; EOQ-5 – Emotional Overeating Questionnaire; EE-3 – Emotional Eating scale; EEQ – Emotional Eater Questionnaire; 95% CI – 95% Confidence Interval for the Spearman's ϱ coefficient.

Supplementary Table S3. Analysis of inter-scale agreement based on discordance (%) between various questionnaire methods for the classification into emotional eating categories in the group of girls studied within the PLACE-19 Study, aged 15-20.

Questionnaire	EES-C	EE-3	EOQ-5
EE-3	36.2	-	-
EOQ-5	38.2	34.4	-
EEQ	37.1	33.6	33.5

EES-C – Emotional Eating Scale for Children and adolescents; EOQ-5 – Emotional Overeating Questionnaire; EE-3 – Emotional Eating scale; EEQ – Emotional Eater Questionnaire.

Supplementary Table S4. Analysis of inter-scale agreement based on discordance (%) between various questionnaire methods for the classification into emotional eating categories in the group of boys studied within the PLACE-19 Study, aged 15-20.

Questionnaire	EES-C	EE-3	EOQ-5
EE-3	41.6	-	-
EOQ-5	45.8	32.8	-
EEQ	38.6	23.7	31.2

EES-C – Emotional Eating Scale for Children and adolescents; EOQ-5 – Emotional Overeating Questionnaire; EE-3 – Emotional Eating scale; EEQ – Emotional Eater Questionnaire.

Supplementary Table S5. Analysis of inter-scale agreement based on Cohen's kappa (95% CI) for comparison between various questionnaire methods for the classification into emotional eating categories in the group of girls studied within the PLACE-19 Study, aged 15-20.

Questionnaire	EES-C	EE-3	EOQ-5
EE-3	0.271 (0.209-0.333)		-
EOQ-5	0.116 (0.043-0.189)	0.307 (0.245-0.368)	-
EEQ	0.255 (0.189-0.322)	0.298 (0.234-0.363)	0.330 (0.265-0.394)

EES-C – Emotional Eating Scale for Children and adolescents; EOQ-5 – Emotional Overeating Questionnaire; EE-3 – Emotional Eating scale; EEQ – Emotional Eater Questionnaire.

Supplementary Table S6. Analysis of inter-scale agreement based on Cohen's kappa (95% CI) for comparison between various questionnaire methods for the classification into emotional eating categories in the group of boys studied within the PLACE-19 Study, aged 15-20.

Questionnaire	EES-C	EE-3	EOQ-5
EE-3	0.156 (0.064-0.249)	-	-
EOQ-5	0.078 (-0.031-0.187)	0.233 (0.126-0.341)	-
EEQ	0.217 (0.121-0.314)	0.336 (0.215-0.458)	0.286 (0.177-0.395)

EES-C – Emotional Eating Scale for Children and adolescents; EOQ-5 – Emotional Overeating Questionnaire; EE-3 – Emotional Eating scale; EEQ – Emotional Eater Questionnaire.

Supplementary Table S7. Analysis inter-scale agreement based on McNemar's test with a test matrix data for comparison between various questionnaire methods for the classification into emotional eating categories in the group of girls studied within the PLACE-19 Study, aged 15-20.

**Questionnaire**	**EES-C**			**EE-3**			**EOQ-5**		
	p	**b (A+, B−)**	**c (A−, B+)**	*p*	**b (A+, B−)**	**c (A−, B+)**	*p*	**b (A+, B−)**	**c (A−, B+)**
EE-3	<0.0001	217	78	-	-	-	-	-	-
EOQ-5	0.9549	155	157	<0.0001	70	211	-	-	-
EEQ	0.0290	171	132	<0.0001	87	187	0.0155	157	116

EES-C—Emotional Eating Scale for Children and adolescents; EOQ-5—Emotional Overeating Questionnaire; EE-3—Emotional Eating scale; EEQ—Emotional Eater Questionnaire; b (A+, B−)—number of participants classified as emotional eaters by questionnaire A and not emotional eaters by questionnaire B; c (A−, B+)—number of participants classified as not emotional eaters by questionnaire A and emotional eaters by questionnaire B.

Supplementary Table S8. Analysis inter-scale agreement based on McNemar's test with a test matrix data for comparison between various questionnaire methods for the classification into emotional eating categories in the group of boys studied within the PLACE-19 Study, aged 15-20.

**Questionnaire**	**EES-C**			**EE-3**			**EOQ-5**		
	p	**b (A+, B−)**	**c (A−, B+)**	*p*	**b (A+, B−)**	**c (A−, B+)**	*p*	**b (A+, B−)**	**c (A−, B+)**
EE-3	<0.0001	106	22	-	-	-	-	-	-
EOQ-5	0.0042	88	53	<0.0001	26	75	-	-	-
EEQ	<0.0001	96	23	0.2418	31	42	0.0002	67	29

EES-C—Emotional Eating Scale for Children and adolescents; EOQ-5—Emotional Overeating Questionnaire; EE-3—Emotional Eating scale; EEQ—Emotional Eater Questionnaire; b (A+, B−)—number of participants classified as emotional eaters by questionnaire A and not emotional eaters by questionnaire B; c (A−, B+)—number of participants classified as not emotional eaters by questionnaire A and emotional eaters by questionnaire B.
